# Parity-dependent association between *TNF*-*α* and *LTF* gene polymorphisms and clinical mastitis in dairy cattle

**DOI:** 10.1186/1746-6148-9-114

**Published:** 2013-06-11

**Authors:** Katarzyna Wojdak-Maksymiec, Joanna Szyda, Tomasz Strabel

**Affiliations:** 1 Faculty of Biotechnology and Husbandry, Department of Animal Genetics and Breeding, West Pomeranian University of Technology in Szczecin, ul. Doktora Judyma 6, 71-466 Szczecin, Poland; 2 Wroclaw University of Environmental and Life Sciences, Kozuchowska 7, 51-631 Wroclaw, Poland; 3 Poznan University of Life Sciences, Wolynska 33, 60-637 Poznan, Poland

**Keywords:** Mastitis, Tumor necrosis factor, Lactoferrin, Immunity, Inflamm-ageing

## Abstract

**Background:**

One major problem in dairy cattle husbandry is the prevalence of udder infections. In today’s breeding programmes, top priority is being given to making animal evaluation more cost-effective and reliable and less time-consuming. We proposed tumor necrosis factor α (*TNF*-α), lactoferrin (*LTF*) and macrophage-expressed lysozyme (*mLYZ*) genes as potential DNA markers in the improvement of immunity to mastitis.

This study included 588 Polish Holstein-Friesian cows kept on one farm located in the north-western region of Poland. All clinical cases of mastitis in the herd under study were recorded by a qualified veterinarian employed by the farm. The following indicators were applied to determine udder immunity to mastitis in the cows under study: morbidity rate (MR), duration of mastitis (DM) and extent of mastitis (EM). *TNF*-α, *mLYZ* and *LTF* genotypes were identified by real-time PCR method, using SimpleProbe technology. Due to the very low frequency of *mLYZ* allele T, the gene was excluded from further analysis.

A statistical analysis of associations between *TNF*-α and *LTF* genes and immunity to mastitis were performed using three models: 1) a parity-averaged model including only additive effects of the genes; 2) a parity-averaged model including both additive and epistatic effects of the genes; and 3) a parity-specific model including only additive effects of the genes.

**Results:**

With the first and second models it was revealed that the genes effects on the applied indicators of immunity to mastitis were non-significant whereas with the third one the effects were found to be statistically significant. Particularly noteworthy was the finding that the effects of *TNF*-α and *LTF* varied depending on age (parity). The alleles which were linked to high immunity to mastitis in lower parities appeared to be less favourable in higher parities.

**Conclusions:**

These interactions might be related to inflamm-ageing, that is an increased susceptibility to infection due to immune system deregulation that progresses with age. Such pattern of interactions makes it impossible to use the genes in question in marker-assisted selection aimed at reducing heritable susceptibility to mastitis. This is because the immune mechanisms behind resistance to infections proved to be too complex.

## Background

One major problem that dairy cattle farmers face is the prevalence of udder infections. Despite considerable technological advancements in animal husbandry, udder inflammation is still widespread, particularly in high-yielding herds. Identification of marker sequences related to immunity to mastitis may be instrumental in improving this trait and therefore reducing the costs associated with the prevention and treatment of the disease. In the case of low heritability traits (such as immunity to mastitis) which have high negative genetic correlation with other traits being subject to selection, marker-assisted selection (MAS) programmes are more effective than traditional methods [1].

Due to their biological functions, *TNF*-α, *LTF* and *mLYZ* genes have been proposed as potential DNA markers for immunity to mastitis. The genes are located in the chromosome regions which have been shown by some authors to contain loci linked to susceptibility to udder inflammation [2-4].

TNF-α is one of the main pro-inflammatory cytokines involved in the immune response. Along with other factors, TNF-α stimulates the proliferation, differentiation and activity of many immune system cells: B lymphocytes, T lymphocytes, NK (natural killer) lymphocytes and LAK (lymphokine-activated killer) cells [5]. It exerts chemotactic and activating effects on monocytes, macrophages and eosinophils, thereby increasing their cytotoxicity. TNF-α also enhances the phagocytic properties of neutrophils, accelerates their release from the bone marrow, and stimulates them to produce reactive oxygen species, which increases their antibacterial and cytotoxic properties. Moreover, TNF-α induces the release of many other cytokines [6]. The gene encoding TNF-α contains four exons and three introns and is located on chromosome BTA23q22 [7]. It is expressed in many types of mammalian cells, but most strongly in macrophages and monocytes. TNF-α production in these phagocytic cells is stimulated by lipopolysaccharide (LPS) found in the bacterial cell wall. In LPS-stimulated macrophages, the expression of *TNF*-α gene triples, the level of mRNA increases approximately 100-fold, while the secretion of the protein itself may be as much as 10,000 times higher [8].

LTF is a multifunctional protein with antimicrobial properties. It is active against many Gram-negative and Gram-positive bacteria, enveloped and non-enveloped viruses, and various types of fungi and parasites [9]. LTF has an ability to bind Fe^3+^ ions [10,11] and other growth agents such as phosphorus and zinc [12]. Thus, availability of chemical elements to potential pathogens becomes limited. LTF is found in plasma, tears, semen, and various mucoserous secretions. However, it is most abundant in neutrophil granules and with them it can be transported to the infection site. Moreover, LTF stimulates neutrophils, the reticuloendothelial (monocyte-macrophage) system and myelopoesis [13]. Thus, it constitutes a vital element of innate immunity, which functions as the organism’s ‘first line of defence’ during an infection [14]. The bovine *LTF* gene has been mapped to chromosome BTA22q24. The coding sequences are divided into 17 exons of 34.5 bp. However, the *LTF* gene expression pattern is markedly different in various mammal species as well as in various tissues and cell types. The expression is regulated via different signal pathways, e.g. by steroid hormones, the growth hormone or the kinase cascade [15].

LYZ is present in most body fluids, the highest concentration being found in tears and chicken egg white. It occurs in the primary and secondary granules of polymorphonuclear granulocytes, and is also produced in monocytes, macrophages and epithelial cells [16]. LYZ is one of the elements of the humoral adaptive immune response. The bactericidal properties of LYZ are primarily due to its destructive effect on the bacterial cell wall: it damages the outer membrane, disintegrates the cytoplasm and increases the permeability of the inner membrane, which eventually leads to bacterial lysis [16,17]. This mechanism is strongest against Gram-positive bacteria whereas Gram-negative strains are less susceptible to LYZ as their cell walls have a more complex structure [18]. The antimicrobial activity of LYZ may also be related to its cationic and hydrophobic properties [18]. LYZ present in different tissues is coded by different genes. The bovine *mLYZ* gene analyzed in this study is an immune-dependent gene. The gene’s structure as well as its organization predispose it to being expressed both in leukocytes (monocytes/macrophages and neutrophils) and in the mammary gland tissue. It consists of four exons and three introns and is located on chromosome BTA5q26 [19]. In intron 2 of this *mLYZ* gene at position 8603, a single nucleotide polymorphism (SNP) (C/T transition) related to cows’ immunity was detected by Pareek *et al*. [20].

## Results

The laboratory analysis showed three genotypes of *TNF*-α and two genotypes of *LTF* and *mLYZ*. Their frequencies are given in Table 1.

**Table 1:** **Frequency of*TNF*-α ,*LTF*and*mLYZ*genotypes and alleles**

**Gene**	**Genotype**	**Number of cows**	**Genotype frequency**	**Allele**	**Allele frequency**
*TNF*-α	*CC*	196	0.3328	*C*	0.5604
	*CT*	267	0.4539	*T*	0.4396
	*TT*	125	0.2133	-	-
*LTF*	*AA*	334	0.5680	*A*	0.7840
	*AB*	254	0.4320	*B*	0.2160
*mLYZ*	*CC*	558	0.9490	*C*	0.9745
	*CT*	30	0.0510	*T*	0,0255
Total	-	588	1.0000	-	1.0000

Due to the very low frequency of *mLYZ* allele *T*, the gene was excluded from further analysis. The effects of the *TNF*-α and *LTF* genes on all the three udder health indicators were found to be non-significant when analyzed for all inter-calving periods taken together. However, quite a different picture arose when the effects were estimated for each period separately.

When analyzing the number of *mastitis* cases with a lactation-averaged model (model 1 and 2), the gene effects were found to be statistically non-significant, whereas when analyzing the interactions between the *TNF*-α gene and parity (model 3), significant effects were observed. In particular, allele *T* of *TNF*-α was associated with a lower number of *mastitis* cases in lower parities and a higher number of *mastitis* cases in higher parities. The associations were confirmed statistically for the second intercalving period (Table 2).

**Table 2:** **The effect of*TNF*-α and*LTF*genes on MR**

**Gene**	**Inter**-**calving period**	**Estimate**	**Standard error**
***TNF***-***α***	1	−0.12230	0.1876
	2	−0.45640*	0.1829
	3	−0.12130	0.2041
	4	0.17480	0.2326
	5	0.12440	0.2640
	6	0.28070	0.3945
***LTF***	1	0.03483	0.3153
	2	0.12500	0.3105
	3	0.25320	0.3364
	4	0.20840	0.3878
	5	0.50650	0.4255
	6	0.88310	0.6460

Asterisks indicate statistical significance levels * P ≤ 0.05.

As for the number of infected udder quarters, no significant associations were found with the parity-averaged models (1 and 2). However, in the case parity-specific estimates (model 3), association was detected between allele *T* of the *TNF*-α gene and a lower number of infected udder quarters, but only in the earlier intercalving periods. A statistically significant effect was observed in the second intercalving period. Similarly, allele *B* of the *LTF* gene was associated with a lower number of affected udder quarters in the first intercalving period whereas in the subsequent periods the same allele had an unfavourable effect (Table 3).

**Table 3:** **The effect of*TNF*-*α*and*LTF*genes on EM**

**Gene**	**Inter**-**calving period**	**Estimate**	**Standard error**
***TNF***-***α***	1	−0.60570	0.3327
	2	−0.83240*	0.3243
	3	−0.57790	0.3619
	4	−0.00314	0.4126
	5	0.42340	0.4683
	6	0.09321	0.6996
***LTF***	1	−0.73600	0.5592
	2	0.10390	0.5507
	3	0.22010	0.5966
	4	0.40560	0.6877
	5	1.18000	0.7546
	6	1.0910	1.1456

Asterisks indicate statistical significance levels * P ≤ 0.05.

The gene x parity interaction was even stronger when related to the number of days with mastitis. The effects of the genes under study proved to be statistically non-significant when averaged over parities (models 1 and 2). On the other hand, an association was found when using a model which included interactions between the genes and parity (model 3). Allele *T* of the *TNF*-α gene was significantly associated with a higher DM, but only in the fifth and sixth intercalving period. Furthermore, allele *B* of the *LTF* gene in the first parity was associated with a lower number while in the fifth one – with a higher number of days with mastitis (Table 4).

**Table 4:** **The effect of*TNF*-*α*and*LTF*genes on DM**

**Gene**	**Inter**-**calving period**	**Estimate**	**Standard error**
***TNF***-***α***	1	−0.05145	0.1109
	2	−0.14070	0.1081
	3	−0.03931	0.1206
	4	−0.06595	0.1375
	5	0.35460*	0.1561
	6	0.67430**	0.2332
***LTF***	1	−0.56560**	0.1864
	2	−0.04408	0.1836
	3	−0.08243	0.1989
	4	0.26440	0.2293
	5	0.58100*	0.2515
	6	0.53880	0.3819

Asterisks indicate statistical significance levels * P ≤ 0.05, ** P ≤ 0.01.

## Discussion

The analysis of the effects of the *TNF*-α and *LTF* genes in relation to clinical mastitis showed strong associations between gene x parity interaction and MR, EM and DM of the disease. Our hypothesis is that these interactions might be related to a phenomenon known as inflamm-ageing.

In humans, ageing is associated with significant changes in the innate immune system such as impairment of the phagocytic activity of neutrophils and macrophages, increased production of pro-inflammatory cytokines and decreased antibacterial defence [21]. The name given to the global age-related dysfunctions of the immune system is immunosenescence [21-23]. The deterioration of the immune system leads to an increased risk of infections such as bacterial infections (pneumonia and urinary tract, skin, and soft-tissue infections) and some viral infections (reactivation of herpes zoster and significantly increased morbidity and mortality due to the influenza virus) [24].

One of the aspects of immunosenescence is “inflamm-aging”, a term coined by Franceschi to refer to a low-grade, chronic and systemic inflammatory status which probably results from a continuous long-term exposure to antigens [25,26]. Many studies on older individuals showed higher LPS-induced production of pro-inflammatory cytokines, particularly IL-1, IL-6, and TNF-α, which are mainly produced by macrophages and fibroblasts [24,27].

The aging process results from a combination of cellular, genetic and environmental factors [28]. In modern theories of aging, a key causative role in this process is attributed to factors that have a direct effect on cells (such as free radicals, metabolic rate, replicative senescence – so-called Hayflick limit, accumulation of harmful metabolites), and to genetic factors (DNA repair defects, accumulation of somatic mutations, or telomere length).

Ageing is not only a major risk factor for infection, but also vice-versa: infection may contribute to the aging process. Franceschi *et al*. [25] suggested three possible models to explain this relationship. First, in the simplest model, direct tissue destruction by a pathogen may play a role in the ageing process. Second, there is possibly a “trade-off” between the capacity of the host defence system to kill pathogens and the damage it causes to the surrounding host tissue. Thus, the beneficial effects of inflammation consisting in the neutralization of pathogenic microbes early in life and in adulthood become detrimental late in life due to an accumulation of damage to the host tissues. In fact, a chronic low-grade inflammation can be seen even in healthy elderly individuals [24]. Third, it is possible that latent or chronic infection contributes to the ageing process. Latent infection might periodically be reactivated, leading to immune-mediated killing of the productively infected cells. Although microorganisms that are able to cause chronic infection usually find ways to avoid immune response, they might contribute to aging through their manipulation of the cell and tissue function [29].

Recent findings suggested that genes involved in the immune response may be particularly prone to age-related changes in DNA methylation [30]. For example, Gowers *et al*. [31] reported age-related loss of methylation of TNF CpG motifs in both peripheral blood leucocytes and macrophages. Lower DNA methylation in the *TNF* promoter may contribute to both an increase in the production of this cytokine and a higher incidence of inflammatory diseases in older individuals. This data suggested that age-related loss of the epigenetic signature of this cytokine may contribute to inflamm-aging.

In the light of the above findings, our concept that significant interactions between the effect of the *TNF*-α gene and parity has to do with inflamm-ageing seems highly probable. In earlier lactations, allele A of the gene encoding TNF-α, whose role is to activate the immune system during infection, proved to be very beneficial – it is linked to higher immunity against infection compared with the other allele in this locus. However, with each successive lactation the effect of the gene becomes increasingly less favourable. The application of the theory of inflamm-aging to interpret the results of this study seems even more justified when we consider the fact that the highest number of statistically significant effects of the *TNF*-α gene was observed for the duration of mastitis. This indicator is most closely related to the chronic status of the disease, which is characteristic of inflamm-aging. Although cows in the sixth or seventh lactation can hardly be considered old, in the case of high-yielding animals the immune dysregulation process is accelerated considerably due to long-term exposure to stressors such as previous infections, mechanical injuries, chemical irritation (*e*.*g*. caused by disinfectants), and strain related to high productivity. The importance of extrinsic factors in the progression of inflamm-ageing has been emphasized, among others, by Larbi *et al*. [32].

The hypothesis that the interaction between the effect of a gene and parity may be associated with inflamm-aging is also justified in the case of the *LTF* gene. This is because LTF, in addition to its bacteriostatic and bactericidal activity, is characterized by anti-inflammatory and anti-allergic properties.

Besides being a natural antibiotic, LTF has immunoregulatory, antineoplastic, and anti-inflammatory functions and plays a role in regulating haematopoiesis [33]. Moreover, LTF is able to bind LPS and soluble CD14, thereby preventing initiation of a CD14-activated proinflammatory expression pattern [34,35]. The protective properties of LTF are due to the fact that it regulates the production and release of cytokines and other factors involved in the inflammatory response: it inhibits pro-inflammatory factors (such as TNF-α, IL-1, IL-8 and histamine) and stimulates anti-inflammatory factors (such as IL-10, IL-4 and IL-6) [34]. Acting as an antioxidant, LTF also protects against oxidative stress. Moreover, LTF has the ability to bind directly to bacterial CpG motifs and thus inhibits their immunostimulating effect [36]. Furthermore, LTF may play an immunoregulatory role and alter the expression pattern in cells via an intracellular signal generated in LTF receptors located on the surface of many cell types (mostly white blood cells – myeloblasts, monocytes, macrophages and lymphocytes, but also epithelial cells) [34].

Perhaps, in the case of the analyzed *LTF* gene variants the above properties of the gene’s product decline with age, which leads to an imbalance between pro- and anti-inflammatory factors. This hypothesis is supported by the results of studies carried out on people). Carrieri *et al*. [37] have shown that centenarians are equipped with gene variants that allow them to optimize the balance between pro- and anti-inflammatory factors and other mediators involved in inflammation. Similarly, Lang *et al*. [38] suggest that research into the capacity of centenarians to exert a protective effect against the adverse outcomes of ageing will help to develop a better understanding of the dysregulation of the balance between pro- and anti-inflammatory pathways.

Our hypothesis is also supported by evolutionary theories of ageing based on antagonistic pleiotropy of the immunity genes [39]. The immune system, by neutralizing pathogens, plays a beneficial role until the time of reproduction and parental care. Subsequently, by causing chronic inflammation, it can have a negative effect late in life, in a period not expected by evolution [39-41]. Age-related diseases are “the price” for an active immune system that defends the body in youth but harms it later in life [42]. Crimmins and Finch [43] even suggest that the genetic polymorphisms responsible for a low inflammatory response in humans might result in a greater chance of longevity.

## Conclusions

Although our study showed significant associations between *TNF*-α and *LTF* and immunity to mastitis, the results would be difficult to apply in marker-assisted selection programmes due to interactions with parity. On the other hand, our findings provide insight into the complex mechanisms of immunity to infections. It is supposed that the interactions between gene effects and parity are related to the phenomenon of inflamm-ageing.

## Methods

### Material

The study was conducted in a herd of approximately 1 000 Polish Holstein-Friesian cows kept on a farm located in the north-western region of Poland. Within this herd, 588 cows were diagnosed at least once with clinical mastitis and only these cows were included in the analyses. An overview of the research material is given in Table 5. All animals lived under similar environmental conditions. They were kept in one free-stall barn and milked twice a day in a herringbone-type milking parlour. The cows had *ad libitum* access to water from individual automatic drinking vessels and were fed an identical standard TMR (total mixed ration) diet. Additionally, during milking each cow was given specially selected feed concentrate suited to its current physiological condition and milk yield. The cows were of different ages and different parities (from first to sixth).

**Table 5:** An overview of the research material

**Inter**-**calving period**/lactation*	**No**. **of cows in herd**	% of cows with mastitis**	**Mean daily milk yield**	**Mean lnSCC for herd**
**I**	813	29.89	30.52	4.89
**II**	918	27.67	34.25	5.14
**III**	868	25.81	35.38	5.47
**IV**	613	29.36	34.89	5.75
**V**	395	35.70	33.60	6.00
**VI**	243	34.98	32.39	6.24
**Total**	990	51.41	31.82	5.58

*The percentage of cows with mastitis was calculated for the whole inter-calving period while the mean daily milk yield and SCC – for the lactation period.

**Cows affected by mastitis at least once in a given inter-calving period.

All clinical cases of mastitis in the herd under study were recorded by a qualified veterinarian employed by the farm. The records specified the number of affected udder quarters and the duration of the disease.

Our study was based on data collected in 2003–2008 for a total of 3,544 lactations, or more specifically inter-calving periods, as the cows were examined both during lactation and in the dry-off period. The average parity per cow amounted to 3.46.

The data was used to analyze not only the number of clinical cases but also the extent and duration of inflammation. The following indicators were applied to determine udder condition/health / immunity to mastitis in the cows under study:

Morbidity rate (MR) – total number of clinical mastitis cases (C) in a particular cow averaged per 100 days of intercalving period (IC) $\mathrm{MR}=\frac{C}{\mathrm{IC}}\times 100$

Duration of mastitis (DM) – total number of days with mastitis (D) in a particular cow averaged per 100 days of intercalving period (IC) $\left(\mathrm{DM}=\frac{D}{\mathrm{IC}}\times 100\right)$

Extent of mastitis (EM) – total number of affected udder quarters (Q) in a particular cow averaged per 100 days of intercalving period (IC) $\mathrm{EM}=\left(\frac{Q}{\mathrm{IC}}\times 100\right)$

The above indicators were calculated separately for each subsequent intercalving period and for all the intercalving periods in total.

### Laboratory method

DNA isolation was performed with ZymoResearch Genomic DNA Kit™ (ZymoResearch, USA) using Zymo-Spin™ IC Fast-Spin column technology. Then, SimpleProbe real-time PCR assays were developed to identify *TNF*-α, *mLYZ* and *LTF* genotypes. The *TNF*-α and *mLYZ* genes (GeneBank Z14137 and U25810, respectively) are known to contain nucleotide transitions C/T, which are responsible for the occurrence of polymorphic forms. On this basis appropriate primers and probes were designed. However, in the case of the *LTF* gene, it is only known that the sixth intron contains a polymorphic site recognized by restrictase *Eco*RI [44]. Therefore, different variants of this gene with regard to the presence of the *Eco*RI polymorphic site were sequenced and a new SNP was found – a *T*/*C* mutation, which was registered in the NCBI dbSNP database (rs109623119). Primers and probes used to identify *TNF*-α, *LTF* and *mLYZ* genotypes are given in Table 6.

**Table 6:** **Primers and probes used to identify*TNF***-***α***, ***LTF*and*mLYZ*genotypes**

***TNF***-***α***	
Forward primer	CCCTTCTCCAGCTGGAAGA
Rewerse primer	ATCTCAGCACTGAGGCGATC
SimpleProbe	CCTGGTACGAACCCAXITCTACCA - PH
***LTF***	
Forward primer	TCATGTTAAGTCACCTGAAATGGTA
Rewerse primer	AGTATGCTGAATATGATACTGGCA
SimpleProbe	CCCAAGTCCATCTATGCATTCCCAG
***mLYZ***	
Forward primer	GCTGAGGAAAGAACAACTAAAATAAT
Rewerse primer	CTTGAGTGATGTCATCTTGCAG
SimpleProbe	CCATCAACAGAXITAAACAGCCCTTAA - PH

PCR reactions were carried out in a LightCycler 2.0 (Roche Molecular Systems Inc., Pleasanton, USA). Each batch consisted of 31 samples and a negative control (water) in 20 μl capillary tubes. The products were analyzed by means of real-time fluorescence readout. Afterwards, a melting curve analysis was performed to detect mutations and examine the product characteristics. Amplification was made with Qiagen® Multiplex PCR Kit (Qiagen GmbH, Hilden, Germany). The PCR mix (10 μl) used to identify *LTF* and *mLYZ* was made up of: 5 μl 2× Qiagen PCR Master Mix (final concentration of 3 mM MgCl_2_); 1 μl each primer (0.2 μM); 1 μl SimpleProbe (0.2 μM); 1 μl water. The temperature profile was as follows: initial denaturation – 95°C for 15min; amplification – 45 cycles: denaturation at 95°C for 20s, annealing at 57°C for 30s, and elongation at 72°C for 40s; melting – 95°C, 40°C and 80°C with ramp rate 0.1°C/min; cooling – 30s.

In the case *TNF*-α, asymmetric real-time PCR was used where the quantity of primer on the same strand as the probe is increased whereas the quantity of the probe is reduced. The composition of the reaction mix was as follows: 5 μl 2× Qiagen PCR Master Mix (final concentration of 3 mM MgCl_2_); 0.5 μl forward primer (0.1 μM); 1.5 μl reverse primer (0.3 μM); 0.5 μl SimpleProbe (0.1 μM); 1.5 μl water. It was necessary to use *Q*-*solution* – a strong coagulant which enhances matrix DNA denaturation thereby preventing primers from forming secondary structures. The following temperature profile was applied: initial denaturation – 95°C for 15min; amplification – 45 cycles: denaturation at 95°C for 30s, annealing at 57°C for 30s, and elongation at 72°C for 60s; melting – 95°C, 40°C and 80°C with ramp rate 0.1°C/min; cooling – 30s.

### Statistical analysis

A zero hypothesis of no effects of the selected gene SNPs on the udder health indicators (*H*_0_ : *q*_*SNP*_ = 0) was examined against an alternative *H*_1_ : *q*_*SNP*_ ≠ 0 using a one-sample t test.

As the values of udder health indicators (MR, EM, or DM) did not have normal distribution, they were transformed into a logarithmic scale.

The following three mixed linear models were applied:

1. A parity-averaged model including only additive effects of the genes:

y=Xq+Zα+e

where:

**y** – vector of trait values defined as ln*T* , where *T* represents a recorded trait (MR, DM, EM), assuming **y** ~ *N*(**Xq**, **ZGZ**^*T*^ + **R**)

**q** – vector of fixed effects (a general mean, a cow’s birth year, additive effects of *TNF*-α and *LTF*);

**α** ~ *N*(0, **G**) vector of random polygenic effects of cows, assuming $G=A\sigma_{\alpha }^{2}$ with **A** representing additive polygenic relationships among individuals, estimated from a pedigree and $\sigma_{\alpha }^{2}$ being a component of the total additive genetic variance;

**e** ~ N(0, **R**) vector of random errors with $R=Ι\sigma_{e}^{2}$ where $\sigma_{e}^{2}$ denotes the error variance;

**X** and **Z** – corresponding design matrices.

2. A parity-averaged model including both additive and epistatic effects of the genes – the same as model 1 plus epistatic (additive by additive) effects.

3. A parity-specific model including gene x parity interaction and the random permanent environmental effect.

Note that the parameterization for the SNPs representing the genes was 0, 1 and 2 for the homozygous, heterozygous and the other homozygous genotype, respectively. All calculations were made using SAS 9.2 software.

## Competing interests

The authors declare that they have no competing interests.

## Authors’ contributions

KWM – concept and design of the paper, collection of data, selection of genes to be analyzed, identification of selected genotypes, interpretation of results, drafting the manuscript and revising it critically for content, final approval of the version to be published. JS – design of the statistical methods, estimation of genes effects, participation in drafting the manuscript. TS – edition, compilation and preparation of data for statistical estimation, participation in drafting the manuscript. All authors read and approved the final manuscript.
